# Penile metastasis of urothelial carcinoma diagnosed by fine-needle aspiration

**DOI:** 10.4103/1742-6413.52832

**Published:** 2009-06-18

**Authors:** Gilda da Cunha Santos, Marcia Lanzoni de Alvarenga, Vinicius Freitas Borlot, Michel Antonio Kiyota Moutinho, Marcello Fabiano de Franco

**Affiliations:** Department of Pathology, Escola Paulista de Medicina, UNIFESP (Federal University of São Paulo)

**Keywords:** Penile metastasis, urothelial carcinoma, fine-needle aspiration

## Abstract

Penile neoplasms are rare and can be primary or represent metastasis or local recurrence. The most common primary cancer of the penis is squamous cell carcinoma, accounting for 95% of all cancers. In spite of the rich vascularity of the organ, penile metastases are uncommon. Cutaneous metastasis of urothelial carcinoma (UC) is extremely rare and generally accepted as the late manifestation of a systemic spread. By 1998, approximately 500 cases of penile metastasis had been reported worldwide. However, only few case reports and series of fine-needle aspiration cytology (FNAC) of penile tumors have been documented. We report a case of penile metastasis from UC diagnosed by FNAC and describe the cytomorphological findings with an emphasis on cercariform cells. Although not commonly used, FNA of penile nodules can be effective in diagnosing recurrence or metastasis and avoiding surgical procedures, thus being an excellent initial procedure in the diagnostic approach.

## CASE REPORT

### Clinical history

A 71-year-old patient presented with a palpable nodule on the penis shaft noted 2 weeks previously and gradually increasing in size. On clinical examination, a nonpainful, firm, nontender nodule measuring 1.0 cm, without ulceration of the overlying skin was identified. He had undergone radical cystectomy 4 months before for urothelial cell carcinoma of the bladder and was referred to the fine-needle aspiration (FNA) clinic for the aspiration of the nodule.

### FNA procedure and cytologic findings

An FNA was performed by a cytopathologist with a 25-gauge needle according to the technique described by Zajicek.[1] The procedure was very well tolerated with little discomfort for the patient. There were no complications. The material was smeared on slides for cytologic interpretation, with some of the slides being air dried and some being fixed in 95% alcohol. The air-dried smears were stained by May-Grünwald-Giemsa and the alcohol-fixed slides were stained by Papanicolaou technique.

**Figure 1:**
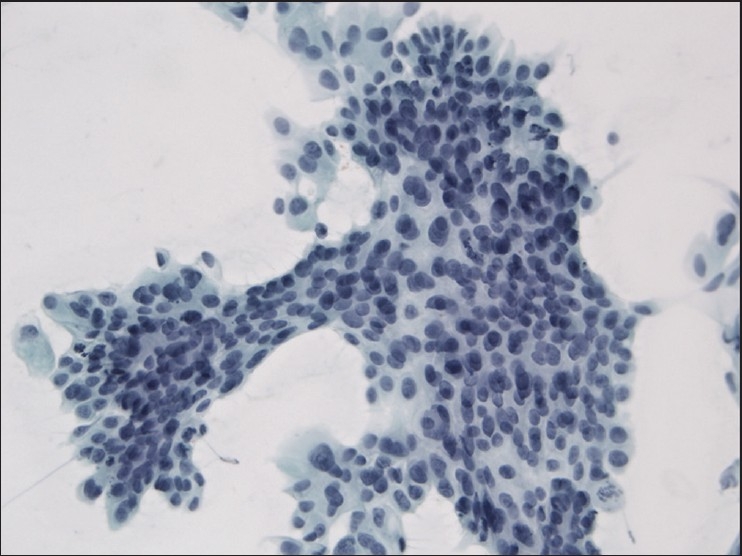
Syncytial cluster of neoplastic cells with abundant basophilic cytoplasm and large hyperchromatic nuclei. Papanicolaou stain, ×400

**Figure 2:**
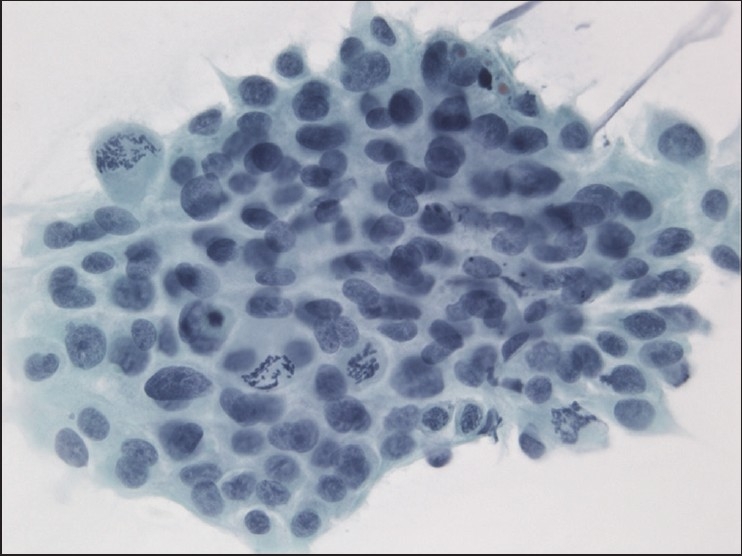
Group of neoplastic cells with frequent mitotic figures. Papanicolaou stain, ×400

**Figure 3:**
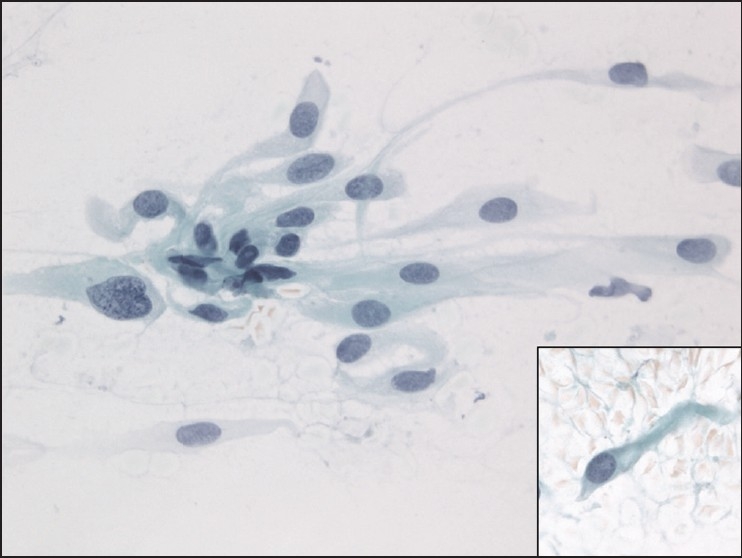
Cercariform cells showing a globular body with eccentric nuclei and a long, thin, unipolar cytoplasmic process. Papanicolaou stain, ×400

**Figure 4:**
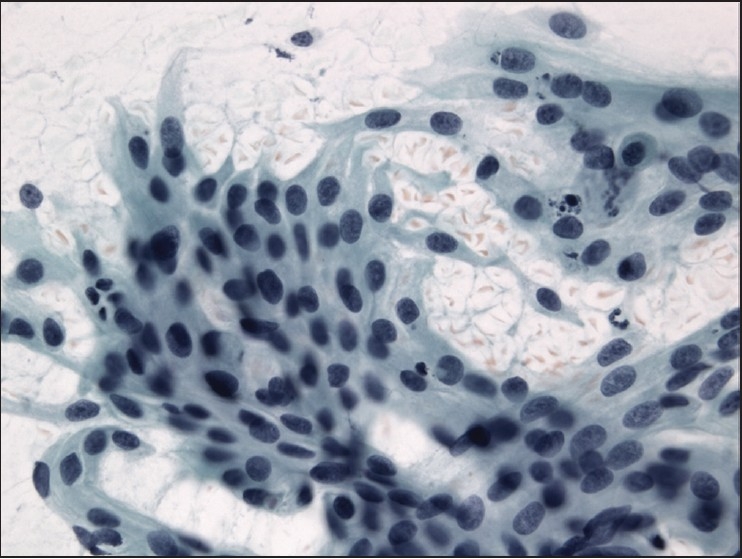
Columnar and cercariform cells demonstrating a fishtail-like end. Papanicolaou stain, ×400

**Figure 5:**
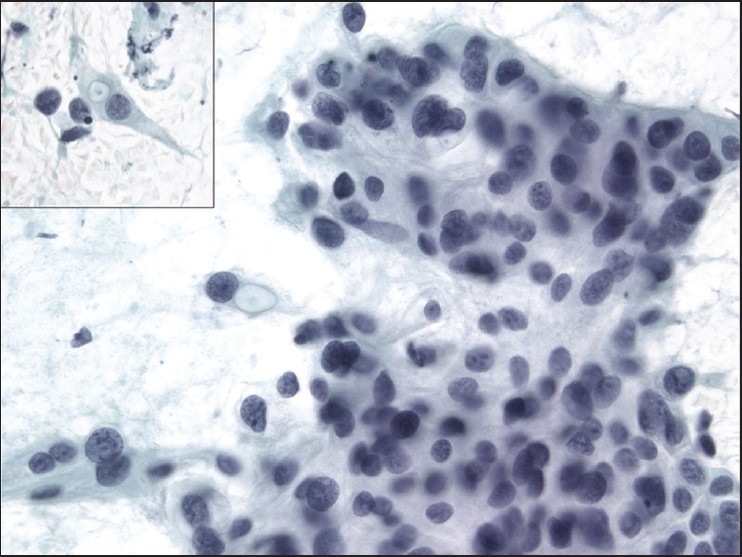
Cercariform cells with small cytoplasmic vacuoles. Papanicolaou stain, ×400

**Figure 6:**
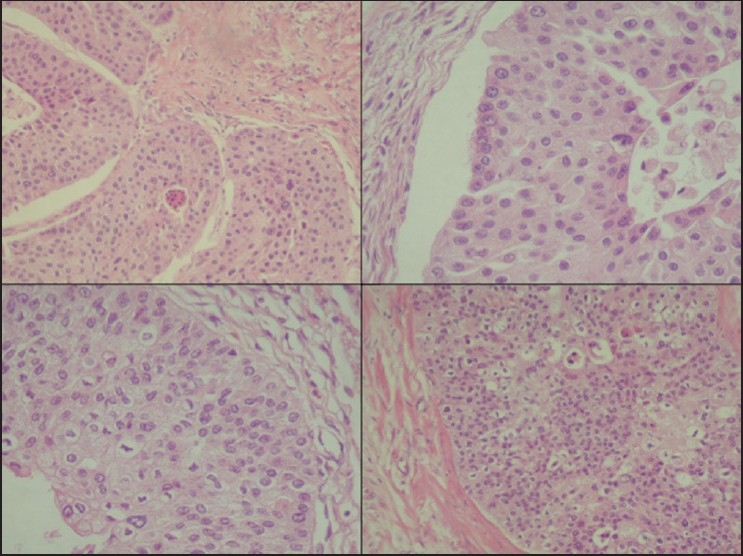
Histologic sections of the cystectomy specimen showing urothelial carcinoma. H&E staining, ×200

The slides showed a highly cellular aspirate consisting of loosely cohesive groups of tumor cells with cuboidal, round, or columnar shape with a dense, basophilic, tapered cytoplasm and large hyperchromatic nuclei [Figure 1]. There were some mitotic figures [Figure 2]. Keratinized cells and papillary formations were not observed. Some cells exhibited a spindle-shaped cytoplasm, eccentric nuclei, and a cytoplasmic process with the appearance of cercariform cells (CCs) [Figures 3 and 4]. Small vacuoles in the cytoplasm were also identified [Figure 5]. A review of the H&E slides of the surgical specimen of the primary bladder tumor showed urothelial carcinoma (UC) [Figure 6].

## DISCUSSION

Although penile metastases are rare, the most common site of origin is the genitourinary system, and within this category, the urinary bladder.[2,3] Cutaneous metastases from primary UC are also extremely rare and are generally accepted as late manifestations of systemic spread.[4]. The route of tumor metastasis to the penis remains unclear but the mechanisms proposed are retrograde venous spread, retrograde lymphatic spread, direct spreading via the arteries, spread by means of implantation and use of instruments, and direct extension.[5]

Recurrence of penile cancer after primary surgery and penile metastasis often present as a nonulcerative nodule. Other manifestations of penile metastasis include priapism, urinary symptoms (urethral hemorrhage, hematuria, incontinence, and irritative and obstructive symptoms), pain, and retention.[6]

It has been argued that the merit of core-needle biopsy lies in its more reliable assessment of the presence of malignant cell infiltration, as well as its histologic extent.[6] However, for the investigation of suspected metastatic disease or recurrence, the identification of a lesion as malignant is usually sufficient for clinical purposes. In this respect, FNAC is used as the initial diagnostic modality in many body sites but it has rarely been used as a diagnostic tool in penile nodules with only few cases and series reported.[7–15] Aspiration cytology should be performed to achieve a diagnostic approach to these penile lesions since it can minimize patient discomfort, and avoids invasive procedures such as incisional biopsies.

Assessment of specific differentiation in a metastatic lesion and a probable site of origin are frequently requested in order to develop effective chemotherapy protocols for treatment and possible surgical resection of the primary tumor. Although the material obtained by FNA exhibited cytomorphologic features characteristic of the primary, already known neoplastic site, the presence of CCs was useful to confirm the urothelial origin. This appearance has been described as a distinct cytomorphologic clue that helps in the diagnosis of metastatic transitional cell neoplasms.[16] The characteristic cell has a nucleated globular body and a cytoplasmic process with a nontapering, flattened, bulbous, or fishtail-like end. Because CCs can also occur in a significant number of squamous cell carcinomas, their diagnostic value is limited as a single variable.[17] Other features described in transitional cell carcinoma include the presence of spindle-shaped, pyramidal, and/or racquet-shaped malignant cells containing eccentrically placed nuclei. A cell population containing features of both squamous and glandular differentiation also favors a diagnosis of metastatic UC.[18] Some authors believe that CCs are a reproducible characteristic of UCs rather than the result of smearing artifact. Despite the preparation method when CCs are not identified in FNA material it is unlikely that the specimen represents metastatic UC.[19] Others extended the definition of CCs to include cells with shorter, broader tails associated with flattened ends. In addition, a small vacuole in the bulbous tail also represented a criterion for identifying CCs.[20] Using a combination of five cytological findings, metastases of UCs were characterized by high rates of CCs and multiple nucleoli and low rates of waxy metaplastic cytoplasm and columnar cells.[17]

Ancillary techniques such as immunocytochemistry can be performed on cytological smears not only to characterize the histological type of the neoplasm, but also to discriminate the primary site of origin.

## CONCLUSIONS

In summary, although not commonly used, FNA of penile nodules can be effective, safe, and highly accurate in diagnosing recurrence or metastasis, especially in nonulcerated lesions. It can minimize patient discomfort, and avoids surgical procedures, thus being an initial choice for diagnostic approach. For the diagnosis of cutaneous metastatic UC, usually a manifestation of systemic spread, the presence of CCs in fine-needle aspirates is a useful morphologic clue.

## COMPETING INTEREST STATEMENT BY ALL AUTHORS

No competing interest to declare by any of the authors.

## AUTHORS' CONTRIBUTIONS

Each author has participated sufficiently in the work and take public responsibility for appropriate portions of the content of this article.

GCS conceived the study, performed the FNAC, participated in its design and coordination, helped in drafting, and scrutinized the manuscript.

MLA participated in the diagnostic workup, performed literature search, and drafted the manuscript.

MKM and VB collected all data and performed the literature search.

MFF participated in the surgical pathology diagnostic workup and scrutinized the manuscript.

This study was conducted with approval from the UNIFESP Ethics Committee. Authors take responsibility to maintain relevant documentation in this respect.

Each author acknowledges that the final version was read and approved.
